# Influence of graft compatibility on fruit quality and mineral nutrient profile in ‘Dinosaur Egg’ (*Prunus domestica × P. armeniaca* ‘Konglongdan’)

**DOI:** 10.3389/fpls.2026.1802338

**Published:** 2026-04-10

**Authors:** Miansen Lei, Yongqiang Huang, Libin Zhang, Sihua Chen, Tianfa Guo, Ming Wang, Xinjian Wang

**Affiliations:** 1 College of Horticulture and Forestry, Tarim University, Alar, China; 2 National and Local Joint Engineering Laboratory of High Efficiency and Superior-Quality Cultivation and Fruit Deep Processing Technology of Characteristic Fruit Trees in Southern Xinjiang, Alar, China; 3 Xinjiang Academy of Forestry, Urumqi, China

**Keywords:** pluot, Siberian apricot, graft compatibility, fruit quality, mineral nutrition, comprehensive analysis

## Abstract

**Introduction:**

‘Dinosaur Egg’ is a premium interspecific hybrid cultivar of pluot, widely cultivated in Xinjiang, China. Its commercial production relies on grafting, for which the Siberian apricot is commonly used as the rootstock. However, this specific graft combination often develops symptoms of incompatibility (such as leaf chlorosis and graft-union cracking), several years after establishment, which ultimately impairs tree growth and fruit quality. To date, a comprehensive understanding of the effects of graft compatibility on the fruit quality and mineral nutrition of ‘Dinosaur Egg’ is still lacking. This study therefore aimed to investigate the influence of different compatibility grades on these traits using the ‘Dinosaur Egg’/Siberian apricot combination, in order to provide a theoretical basis for fruit quality regulation and improved cultivation practices.

**Methods:**

Using eight-year-old ‘Dinosaur Egg’/Siberian apricot grafted plants as experimental material, the trees were categorized into four distinct graft compatibility grades based on leaf and graft-union phenotypes: normal (control, CK), Grade I, Grade II, and Grade III (in descending order of compatibility). A total of 39 parameters encompassing fruit appearance, flavor, bioactive compounds, and mineral nutrition were measured for each grade. The data were subsequently subjected to significance analysis, correlation analysis, principal component analysis, and a comprehensive evaluation based on a membership function model.

**Results:**

The results demonstrated that graft compatibility significantly influenced both fruit quality and mineral nutrient accumulation in ‘Dinosaur Egg’. High compatibility markedly increased fruit size, whereas lower compatibility, despite reducing individual fruit fresh weight, significantly enhanced fruit firmness and soluble solids content, while shifting peel coloration from red to bright yellow. Notably, the highest total soluble solids to titratable acidity ratio was recorded in Grade II fruits, corresponding to superior flavor quality. Furthermore, lower compatibility significantly increased the contents of soluble solids, total phenolic, and total flavonoid, but decreased total anthocyanin content. In terms of mineral nutrition, fruits under low-affinity conditions accumulated higher concentrations of B, P, K, and Mg, but lower concentrations of Fe, Mn, Cu, and Zn. Following correlation analysis, which reduced the 39 initial quality indicators to 20 key parameters, principal component analysis then categorized these parameters into five distinct dimensions. Finally, a comprehensive evaluation based on these dimensions yielded a fruit quality ranking of Grade II > CK > Grade III > Grade I.

**Discussion:**

Moderately low graft compatibility triggered a beneficial hormetic response, facilitating an optimal balance across multiple quality dimensions instead of the maximization of any single trait. Thus, this study demonstrates that pursuing an optimal, rather than the highest possible, compatibility represents a novel and effective strategy for enhancing the integrated quality of ‘Dinosaur Egg’ fruit.

## 1 Introduction

The pluot (*Prunus domestica × Prunus armenica*) is a stone fruit tree belonging to the genus Prunus in the family Rosaceae. It is a novel hybrid fruit that was selected and bred in the 1990s through repeated crosses between plum (*Prunus domestica* L.) and apricot (*Prunus armeniaca* L.) by American fruit tree breeders (Ahmad et al., 2004; Salava and Brožová, 2024). The pluot, a novel interspecific hybrid, combines the distinctive flavors of apricot and plum. It is recognized as a remarkable fruit innovation of the 21st century (Aning et al., 2025). By 2025, the total cultivation area of pluots in China has reached approximately 20,000 hectares. Xinjiang accounts for about 70% of this area, and national planting continues to expand. Among the seven introduced pluot varieties, ‘Dinosaur Egg’ (*Prunus domestica × Prunus armeniaca* ‘Konglongdan’) is particularly notable for its attractive appearance—characterized by large fruit size and bright coloration—and its excellent eating quality, featuring thin skin, thick flesh, exceptional sweetness, juiciness, and a rich aroma (Crisosto et al., 2007; Lei et al., 2026). Furthermore, it is a rich source of essential mineral nutrients (e.g., phosphorus, potassium, calcium, magnesium, iron) and bioactive compounds, including carotenoids, flavonoids, and phenolic substances (Wills et al., 1983; Tomás-Barberán et al., 2001; Milani et al., 2017; Popova et al., 2023). To maintain its superior fruit quality and ensure efficient cultivation, propagation primarily relies on grafting techniques (Baron et al., 2019).

Grafting is a traditional yet crucial technique for plant propagation and improvement, and its success hinges on graft compatibility, which refers to the characteristics of functional vascular bundle connection and physiological integration between the rootstock and the scion (Dogra et al., 2018; Mauro et al., 2022). Graft incompatibility, a major horticultural constraint, is characterized by distinct anatomical abnormalities that impair vascular function and disrupt fruit mineral supply. Anatomically, incompatibility is primarily characterized by impaired callus fusion with disorganized proliferation and persistent isolation layers (Feng et al., 2024), core vascular disorders, asynchronous cambial activity (Melnyk et al., 2015; Wang et al., 2025a), and interface malformations (Loupit et al., 2023). The vascular disorders include phloem sieve tube reduction accompanied by callose deposition (Amri et al., 2021; Wang et al., 2022), as well as malformed xylem vessels (e.g., spiraling tracheary element bundles) with abnormal lignification (Wu et al., 2022). These abnormalities compromise the vascular system: xylem forms discontinuous connections, restricting water and mineral transport (Vadde, 2022), while active phloem transport is significantly inhibited (Moing et al., 1987). These vascular impairments have direct consequences for the mineral nutrient supply to the fruit. Calcium, which is transported almost exclusively through the xylem, is particularly sensitive to such disruption. Studies on incompatible apple rootstocks have shown significant alterations in the distribution of both calcium and potassium across the graft union, directly impacting their availability to the fruit (Simons and Chu, 1983). The transport of other key elements like boron, phosphorus, and potassium is similarly affected (Thompson and Zwieniecki, 2005; Holbrook and Zwieniecki, 2011). When these primary long-distance transport pathways are compromised, fruits acquire minerals via residual xylem (Dražeta et al., 2004), short-distance intercellular transport (apoplastic/symplastic; Yin et al., 2012), scion mineral remobilization (Wang, 2011), and minor direct absorption. However, the relative contribution of these compensatory mechanisms under conditions of graft incompatibility remains to be elucidated. It has been reported that graft incompatibility can manifest in two primary forms beyond complete failure: “translocated” and “localized” incompatibility (Mosse, 1962; Moreno et al., 1993; Errea et al., 2001; Zarrouk et al., 2010; Reig et al., 2018). These forms are not restricted to specific species and may co-occur within a single graft combination. Translocated incompatibility is typified by distinct foliar symptoms, including leaf yellowing that progresses to red or orange discoloration, followed by premature abscission. In contrast, localized incompatibility often results in mechanical failure at the graft union several years post-grafting or under high-wind conditions. This failure is fundamentally attributed to a discontinuity or poor connection in the vascular tissues across the graft interface. The rootstock serves as the foundation of the grafted tree, it plays a critical role not only in water and nutrient uptake but also in regulating scion growth, stress tolerance, and ultimately, fruit yield and quality (Grigolo et al., 2021; Habibi et al., 2022). Therefore, the selection of a compatible rootstock is of paramount importance.

In China, rootstocks commonly used for pluot cultivation include Chinese plum (*Prunus salicina*), Nanking cherry (*Prunus tomentosa*), Siberian apricot (*Armeniaca sibirica* (L.) Lam.), David peach (*Prunus davidiana*), and peach (*Prunus persica*). Among these, Siberian apricot—a shrub or small tree widely distributed across northern China, exhibits traits including cold hardiness, drought tolerance, and resilience to saline-alkaline and poor soils (Jian et al., 2022). Owing to these adaptive advantages, it has become the predominant rootstock for pluot grafting. However, in field production, ‘Dinosaur Egg’ pluot trees grafted onto Siberian apricot rootstocks often exhibit delayed graft incompatibility. Symptoms—including leaf chlorosis, swelling, and cracking at the graft union—typically begin to manifest 4–5 years after grafting. In severe cases, this incompatibility can lead to structural weakness, resulting in trunk breakage or tree loss under windy conditions. These symptoms align with those described as “translocated” graft incompatibility in apricot, plum, and peach grafting systems by Reig et al. (2018; 2019), Moreno et al. (1993), and Hartmann et al. (1990). This condition can be diagnosed through assessment of leaf coloration, overall tree vigor, and anatomical integrity of the graft union.

For the pluot, fruit quality—encompassing appearance, flavor, nutritional value, and bioactive compounds—is a primary determinant of its commercial value and market competitiveness (Gull et al., 2022). However, current understanding of how graft compatibility shapes fruit quality is largely derived from studies on crops such as cherry (Martins et al., 2021), pear (Çoban and Öztürk, 2022), watermelon (Liu et al., 2025), kiwifruit (Li et al., 2021), cucumber (Al-Debei et al., 2025), and melon (Camalle et al., 2023). Systematic investigations focusing specifically on the pluot remain scarce. The regulatory role of graft compatibility in determining fruit quality parameters has been well-documented in these established systems. For instance, research on various horticultural crops, including grape (Jia et al., 2025; Monteiro et al., 2025; Yin et al., 2025), tomato (Flores et al., 2010), citrus (Morales et al., 2021; Qureshi et al., 2021), eggplant (Ulas, 2021), and melon (Camalle et al., 2023), has demonstrated that rootstock-scion compatibility significantly regulates plant growth, yield, and the metabolism and accumulation of key fruit quality components such as sugars, organic acids, amino acids, and polyphenols. Collectively, these findings underscore that rootstock selection and compatibility assessment are critical for premium fruit production.

To date, limited information is available on how local translocation incompatibility in ‘Dinosaur Egg’ pluot grafted onto Siberian apricot rootstocks affects comprehensive fruit quality. Therefore, this study used ‘Dinosaur Egg’ pluot/Siberian apricot combinations as plant materials. Based on morphological symptoms observed in the field, the trees were categorized into four compatibility grades: Normal (Control, CK), Grade I, Grade II, and Grade III (with compatibility decreasing sequentially). We systematically measured and analyzed the appearance, flavor, mineral nutrients, and bioactive compounds in fruits from each grade. The objectives of this study were to: (i) investigate how graft compatibility grades affect the commercial attributes, flavor profiles, and nutritional composition of ‘Dinosaur Egg’ pluot fruit; (ii) characterize the differences in mineral nutrient concentrations among the different compatibility grades; and (iii) identify the optimal compatibility grade for superior fruit quality through a comprehensive evaluation. This work aims to elucidate the intrinsic relationship between graft compatibility and fruit quality traits along with mineral nutrition, thereby advancing the theoretical understanding of stress-induced quality optimization and providing practical guidance for rootstock selection to enhance pluot fruit quality in commercial production.

## 2 Materials and methods

### 2.1 Plants and sample preparation

The field trial was conducted in Wensu County, Aksu Prefecture, Xinjiang Uygur Autonomous Region, China (80°11′37″ N, 41°24′36″ E; elevation: 1301 m). The site features a typical warm-temperate continental arid climate, characterized by an annual sunshine duration of 2800–3000 h, a mean annual temperature of 10.1 °C, an extreme minimum temperature of −27.6 °C, a mean annual precipitation of 42.4–94 mm, a mean annual evaporation of 1948 mm, and a frost-free period of 180–227 days. Situated on an alluvial fan plain at the southern foot of the Tianshan Mountains, the terrain is flat and open. The soil is classified as brown desert soil with the following properties: pH, 7.33; total salt content, 0.11%; organic matter, 0.39%; available phosphorus, 12.87 mg·kg^-1^; available potassium, 184.82 mg·kg^-1^; and alkali-hydrolyzable nitrogen, 13.68 mg·kg^-1^. According to soil nutrient grading standards for fruit tree cultivation in China, available phosphorus of 10–20 mg·kg^-1^ is considered adequate for normal growth of temperate fruit trees, while available potassium above 150 mg·kg^-1^ is considered rich or moderately rich. Therefore, both phosphorus and potassium supplies in the experimental soil are sufficient for plum (*Prunus*) tree growth.

Eight-year-old ‘Dinosaur Egg’ pluot (*Prunus domestica* × *Prunus armeniaca* ‘Konglongdan’) trees grafted onto Siberian apricot (*Armeniaca sibirica* (L.) Lam.) rootstocks were used as plant materials. The rootstocks were transplanted at one year of age and grafted at two years of age. Trees were planted at a spacing of 3 m × 5 m (row × tree). All field management practices, including irrigation, fertilization, and pest and disease control, followed conventional orchard protocols and were kept uniform across the experimental plot.

**Figure 1:**
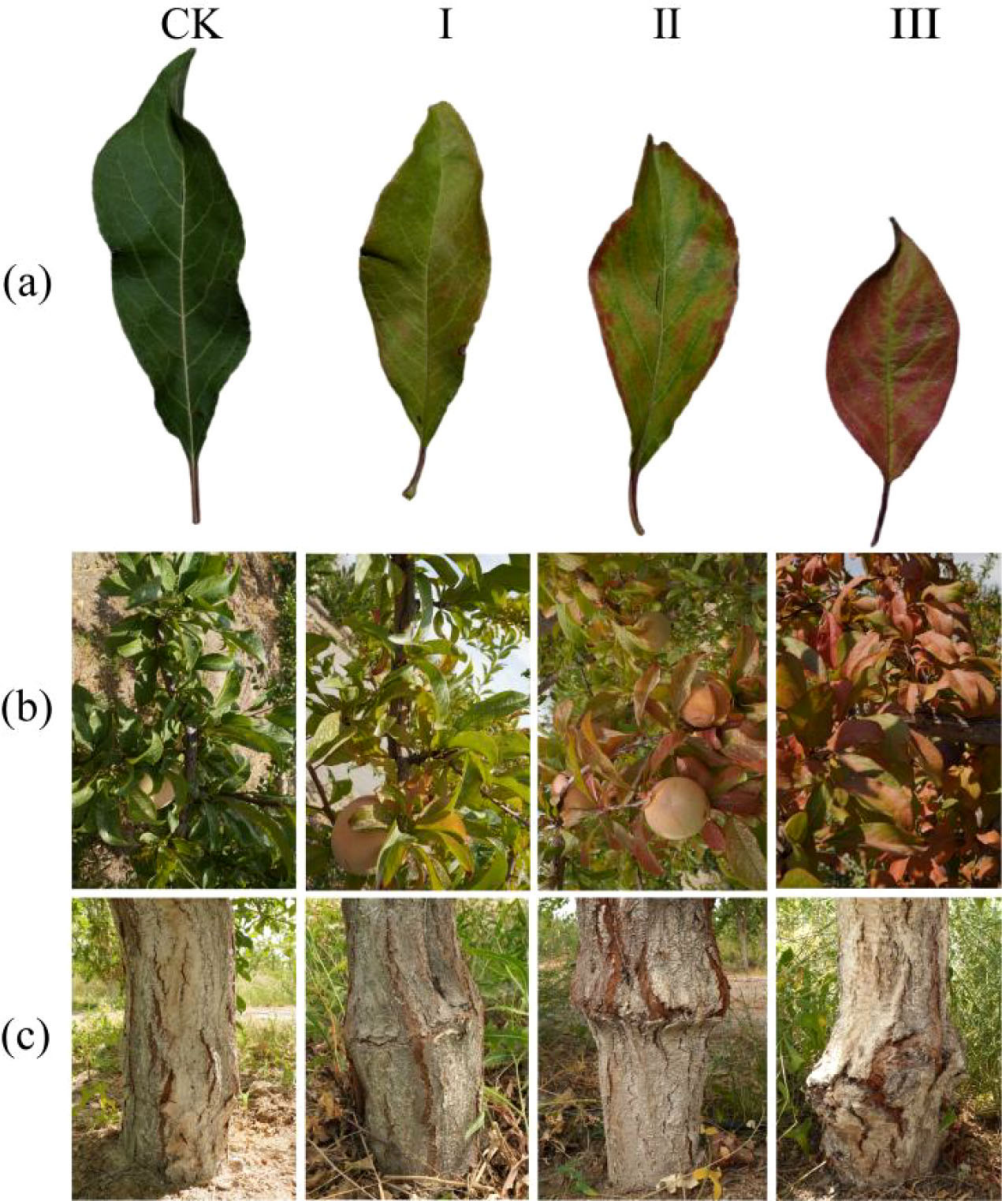
Phenotypic characteristics of ‘Dinosaur Egg’ plants under different graft compatibility grades. CK, graft-compatible combination (control); I–III, combinations in decreasing compatibility (Grade III being the lowest).. **(a)** Single-leaf phenotypes. **(b)** Overall plant leaf phenotypes. **(c)** External view of the graft union.

In August 2024, grafted trees were categorized based on established methods for assessing graft incompatibility in species such as apricot, plum, and peach (Moreno et al., 1993; Errea et al., 2001; Zarrouk et al., 2010; Tang, 2016; Tang et al., 2017; Reig et al., 2018; Amri et al., 2021). Plants exhibiting normal compatibility served as the control (CK). Incompatible plants were subsequently classified into three grades of decreasing compatibility—Grade I, Grade II, and Grade III—according to foliar visual symptoms, specifically color transition (from yellowing to reddening), the Citrus Color Index [ $\text{CCI}=1000{\text{a}}^{*}/({\text{L}}^{*}\cdot {\text{b}}^{*})$] (Villaseñor-Aguilar et al., 2024), and graft union integrity (**Figure 1**, **Table 1**).

**Table 1:** Criteria for grading graft compatibility in the ‘Dinosaur Egg’/Siberian apricot combination based on leaf and graft-union phenotypes.

Graft compatibility grade	Leaf visual diagnosis	Graft union quality	Blade CCI value
CK	Leaves dark green	Texture continuous and smooth, without noticeable callus formation	-13 ≤ CCI < -8
I	Leaves chlorotic (yellow-green).	Texture with minor fissures; slight callus formation	-8 ≤ CCI < 2
II	Leaves exhibiting green-red mosaicism	Texture with evident fractures and moderate local swelling	2 ≤ CCI < 12
III	Leaves predominantly reddish-brown.	Texture completely discontinuous with severe swelling and cracking	12 ≤ CCI < 22

The experiment comprised four treatments (including CK), with each treatment having three independent biological replicates, and each replicate consisting of 10 trees. Fruit sampling was conducted at commercial maturity in August 2025: from each tree, a total of 10 uniformly sized, defect-free fruits were harvested from five canopy positions (east, west, south, north, and center). All samples were immediately transported to the laboratory; upon arrival, they were rinsed sequentially with tap water and deionized water and then gently blotted dry with paper towels. For subsequent destructive measurements, 50 fruits were randomly sampled from each replicate within a treatment, resulting in a total of 150 fruits analyzed per treatment. These fruits were used to determine individual fruit weight, longitudinal diameter, transverse diameter, exocarp color, mesocarp color, fruit firmness, soluble solids content, and ascorbic acid content. For imaging, representative fruits were selected from each treatment and photographed to document top, bottom, and side views, as well as transverse and longitudinal sections. The remaining fruits were peeled, and the edible tissue from the equatorial region was excised, immediately frozen in liquid nitrogen, and homogenized using a laboratory blender. The resulting homogenized sample was stored at −80 °C for subsequent analysis of mineral elements, titratable acidity, soluble sugars, total flavonoids, total phenolics, and others.

### 2.2 Appearance attributes

Fresh weight was determined using a JE520 electronic analytical balance (Shanghai Puchun Measure Instrument Co., Ltd., China) with an accuracy of 0.01 g. Fruit firmness (measured on intact fruit with peel) was assessed using a TA. GEL texture analyzer (Shanghai Baosheng Industrial Development Co., Ltd., China), with results expressed in newtons (N). Longitudinal diameter (L) and transverse diameter (T) were measured using an ABSOLUTE 133-002A digital caliper (Guilin Measuring & Cutting Tool Co., Ltd., China) accurate to 0.01 mm. Color parameters of the exocarp and mesocarp—lightness (L^*^), red–green chromaticity (a^*^), and yellow–blue chromaticity (b^*^)—were measured using an UltraScan PRO colorimeter (HunterLab, USA). Measurements were taken at five equidistant points around the equatorial region of each fruit and averaged. The fruit shape index (FSI) (Jiao et al., 2023) was calculated using Equation 1; chroma (C), hue angle (H, in degrees), and color index of ‘Dinosaur Egg’ (CIDE) (Carreño et al., 1995) were subsequently calculated using Equations 2, 3, and 4, respectively.

(1) FSI=L/T

(2) $$\text{C}=\sqrt{{\left({\text{a}}^{*}\right)}^{2}+{\left({\text{b}}^{*}\right)}^{2}}$$

(3) $$\text{H}=\text{arctan}({\text{b}}^{*}/{\text{a}}^{*})$$

(4) $$\text{CIDE}=(180-\text{H})/({\text{L}}^{*}+\text{C})$$

### 2.3 Physical and chemical indicators

Fruit biochemical traits, including soluble solids content (SSC), total soluble sugars (TSS), titratable acidity (TA), total phenolics, and total flavonoids, were determined following the methods described by Isengard (Isengard, 2001), Wang et al. (Wang et al., 2025b), and Contessa et al (Contessa et al., 2013). Moisture content was determined using a GZX-9420 MBE electric thermostatic drying oven (Shanghai Boxun Medical Biological Instrument Corp., China). Soluble solids content was measured with an RX5000a automatic benchtop refractometer (ATAGO, Japan). Titratable acidity was quantified according to the acid-base neutralization titration method specified in the “National Food Safety Standard—Determination of Total Acids in Food” (GB 12456–2021). Total soluble sugars (TSS) were analyzed by the anthrone-sulfuric acid colorimetric method. The SSC/TA and TSS/TA ratios were then calculated from these measurements. Total phenolics were quantified using the Folin–Ciocalteu method. Ascorbic acid content (vitamin C, VC) was determined by high-performance liquid chromatography according to the “National Food Safety Standard—Determination of Ascorbic Acid in Food” (GB 5009.86–2016). Soluble protein content was assayed using Coomassie Brilliant Blue G-250. Total flavonoids were analyzed by the aluminum chloride colorimetric method. Total anthocyanins were measured by the pH-differential method, and proanthocyanidins by the sulfuric acid–vanillin method. Total carotenoids were determined using an Infinite M200 PRO full-wavelength microplate reader (Tecan, Austria).

### 2.4 Mineral composition

Following the “National Food Safety Standard—Determination of Multiple Elements in Food” (GB 5009.268–2025), fruit samples were digested. The resulting clear digests were analyzed for total phosphorus (P), total potassium (K), calcium (Ca), and magnesium (Mg) using an inductively coupled plasma optical emission spectrometer (ICP-OES; Avio 200, PerkinElmer, USA). Concentrations of iron (Fe), manganese (Mn), copper (Cu), zinc (Zn), and boron (B) were determined using an inductively coupled plasma mass spectrometer (ICP-MS; NexION 1000G, PerkinElmer, USA). The determination results are expressed as mg·kg^-1^ fresh weight (FW).

### 2.5 Statistical analysis

Statistical analyses were performed using SPSS 27.0 (IBM, USA). Differences in fruit quality traits among the four graft compatibility grades (CK, Grade I, Grade II, and Grade III) were evaluated by one-way analysis of variance (ANOVA) followed by Duncan’s multiple range test, with statistical significance set at *P* < 0.05. Pearson correlation analysis was carried out on the online Metware Cloud platform (https://cloud.metware.cn) using the default parameters.

Principal component analysis (PCA) of the fruit quality traits was performed using Origin 2024 (OriginLab Corporation, USA). Subsequently, a comprehensive evaluation of fruit quality across the four graft compatibility groups (CK, Grade I, Grade II, and Grade III) was conducted using the membership function method (Hou et al., 2010; Shen et al., 2014; Jia et al., 2025). Based on the PCA results and the physiological attributes of the traits, the traits were categorized into distinct dimensions. Scores for each treatment group within these dimensions were then calculated. The specific computational procedures were as follows:

Membership function values were derived from Equations 5-7, corresponding to positive indicators (Equation 5), negative indicators (Equation 6), and moderate indicators (Equation 7), respectively.

(5) $${\text{X}}_{\text{ij}}^{'}=\frac{{\text{X}}_{\text{ij}}-\min({\text{X}}_{\text{j}})}{\max({\text{X}}_{\text{j}})-\min({\text{X}}_{\text{j}})}\times \text{α}+(1-\text{α})$$

(6) $$X_{ij}^{'}=\frac{\max(X_{j})-X_{ij}}{\max(X_{j})-\min(X_{j})}\times \alpha +(1-\alpha )$$

(7) $$X_{ij}^{'}=1-\frac{\left|X_{ij}-X_{jopt}\right|}{max(D_{j})}\times (1-\alpha )$$

where ${\text{X}}_{\text{ij}}^{"}$ is the membership function value for the *i* treatment and *j* indicator; ${\text{X}}_{\text{ij}}$ is the corresponding original measurement; $\text{max}({\text{X}}_{\text{j}})$ and $\text{min}({\text{X}}_{\text{j}})$ represent the maximum and minimum values of the *j* indicator, respectively; ${\text{X}}_{\text{jopt}}$ denotes the ideal moderate value for the *j* indicator, defined in this study as the arithmetic mean of all sample values for that indicator; and α is the efficacy coefficient ( α = 0.6).

Weight values were subsequently calculated using Equations 8–10.

(8) $$f_{ij}=\frac{X_{ij}^{'}}{\sum_{i=1}^{m}X_{ij}^{'}}(i=1,\;2,\ldots ,m;j=1,\;2,\ldots ,n)$$

(9) $$H_{j}=-\frac{1}{\ln m}\sum_{i=1}^{m}f_{ij}\ln f_{ij}$$

(10) $$W_{j}=\frac{1-H_{j}}{n-\sum_{j=1}^{n}H_{j}}$$

where ${\text{f}}_{\text{ij}}$ is the proportion of the *i* treatment for the *j* indicator, ${\text{H}}_{\text{j}}$ denotes the entropy of the *j* indicator, and ${\text{W}}_{\text{j}}$ represents the corresponding weight.

(11) $$R_{i}=\sum_{j=1}^{n}W_{j}\cdot X_{ij}^{'}$$

The composite evaluation score (*R_i_*) was calculated using Equation 11. All treatments were then ranked in descending order based on their *R_i_* values, with a higher score indicating superior overall fruit quality.

(12) Dik=∑j∈Sk(Xij'×Wj)

The dimensional score for each treatment was computed using Equation 12, where *D_ik_* denotes the score of the *i* treatment in the *k* quality dimension, *S_k_* is the set of indicators belonging to the *k* dimension, ${\text{X}}_{ij}^{'}$ is the membership value of the *i* treatment for the *j* indicator, and *W_j_* is the weight of the *j* indicator.

## 3 Results

### 3.1 Analysis of fruit quality

#### 3.1.1 Morphological and physical attributes of fruits

**Figure 2:**
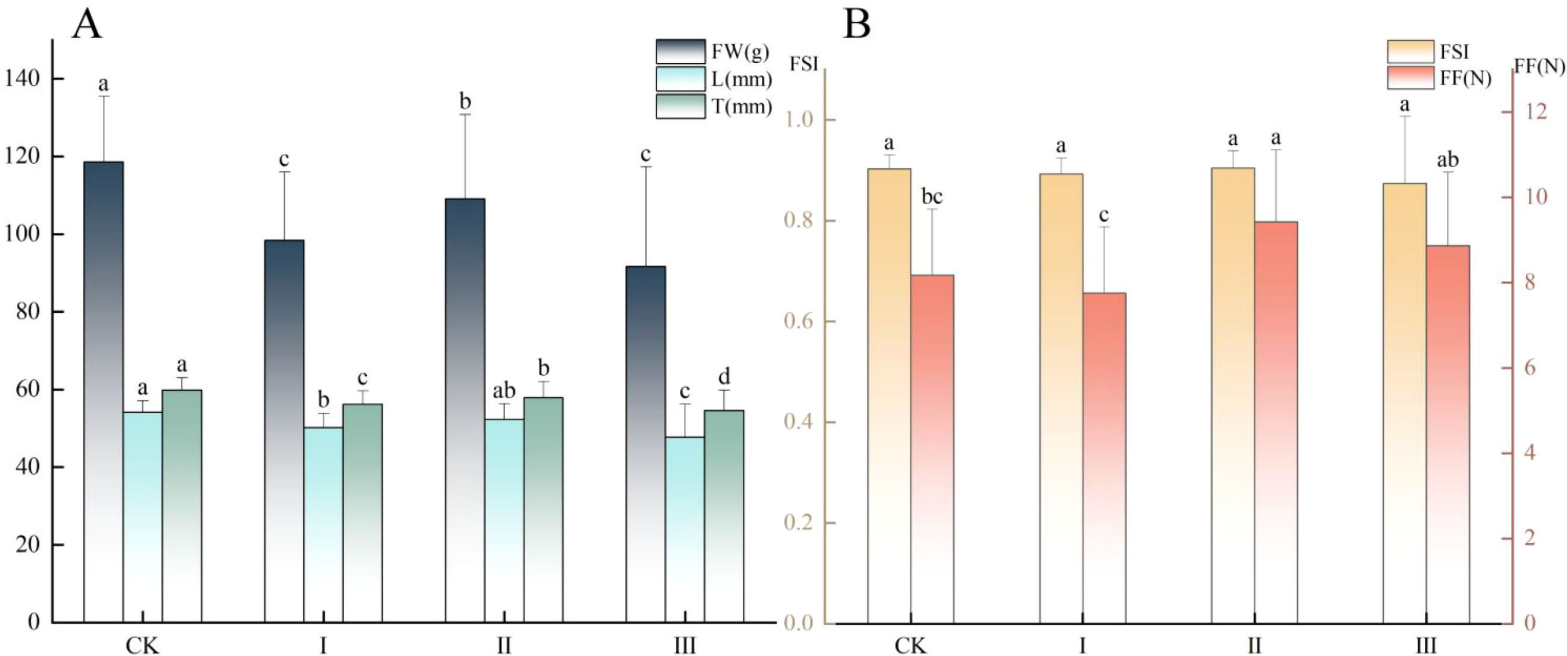
Fruit morphology and physical properties of ‘Dinosaur Egg’ across graft compatibility grades. **(A)** Fresh weight (FW), longitudinal diameter (L), and transverse diameter (T); **(B)** Fruit shape index (FSI) and fruit firmness (FF). Data are presented as mean ± SD (n = 150), Standard Deviation (SD). Different lowercase letters (a–d) above bars indicate statistically significant differences among means within each measured parameter at *P* < 0.05, as determined by Duncan’s multiple range test.

Graft compatibility significantly influenced the fresh weight, longitudinal diameter, transverse diameter, and firmness of ‘Dinosaur Egg’ fruits (*P* < 0.05; **Figure 2**), whereas no significant differences were detected in the fruit shape index among the treatments (**Figure 2B**). Among the four compatibility groups, fruits from the control exhibited the highest values for fresh weight, longitudinal diameter, and transverse diameter (**Figure 2A**). Specifically, the fresh weight of the control fruits was significantly greater than that of fruits from Grade I, II, and III. For longitudinal diameter, the control was comparable to Grade II but significantly larger than Grades I and III. The transverse diameter of the control fruits was significantly larger than that of all grafted treatment groups (Grades I-III). In contrast, fruit firmness showed an opposite trend (**Figure 2B**). Fruits from Grades II and III had significantly higher firmness than those from the control and Grade I, with Grade I fruits displaying the lowest firmness. In summary, higher graft compatibility (CK) promoted fruit expansion and morphological development, while lower compatibility (Grades II and III) was associated with increased fruit firmness.

#### 3.1.2 Fruit color traits

**Figure 3:**
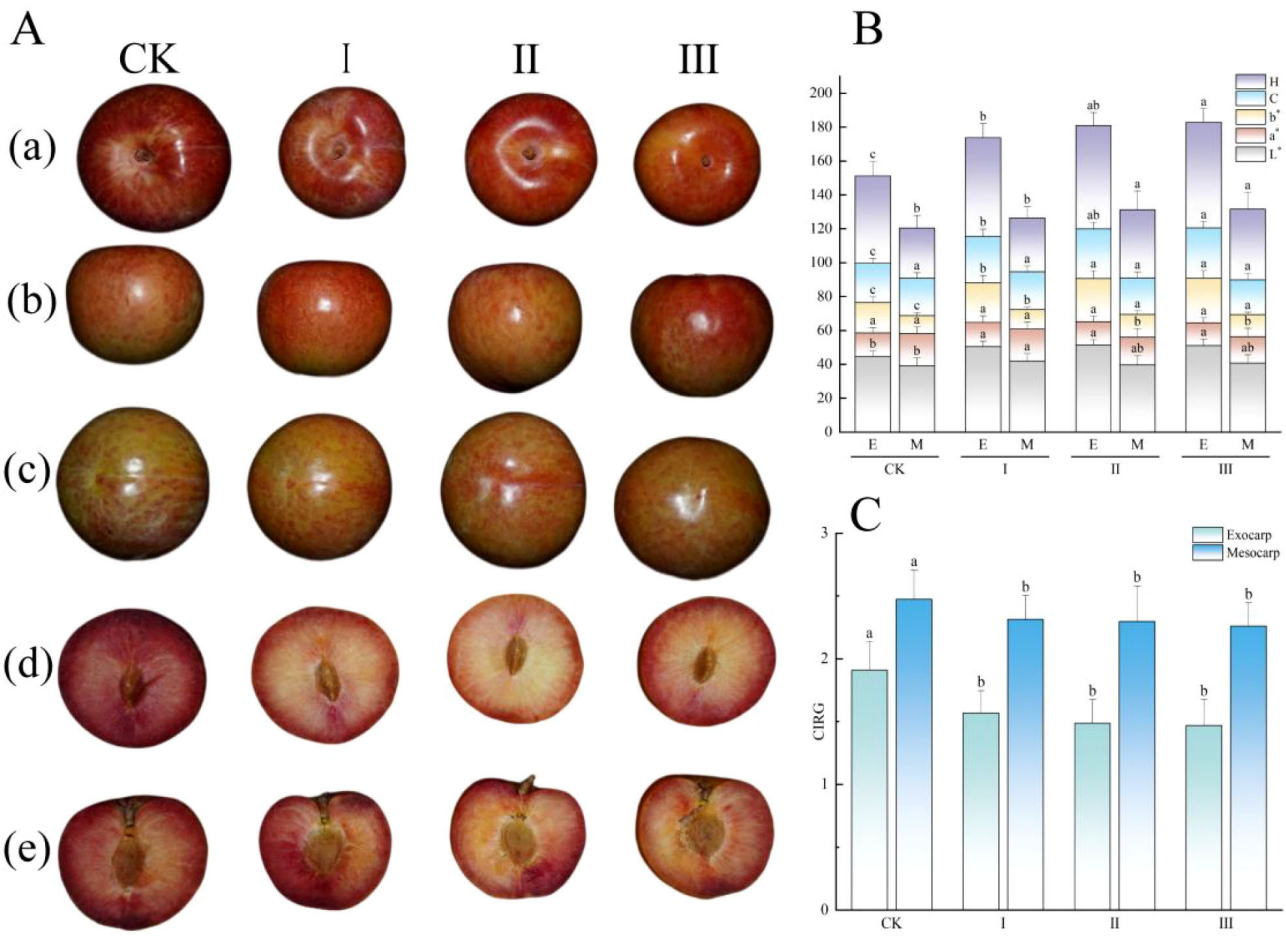
Morphological and colorimetric traits of ‘Dinosaur Egg’ fruits across graft compatibility grades. **(A)** Fruit morphological characteristics. (a) Lateral view with the stem end upward. (b) Lateral view with the longitudinal axis horizontal. (c) Lateral view with the stem end downward. (d) Transverse section. (e) Median longitudinal section. **(B)** Fundamental color parameters of the exocarp (E) and mesocarp (M): lightness (L^*^), red–green chromaticity (a^*^), yellow–blue chromaticity (b^*^), chroma (C), and hue angle (H). **(C)** Color index of ‘Dinosaur Egg’ (CIDE) for the exocarp and mesocarp. Data are presented as mean ± SD (n = 9). Different lowercase letters (a–d) above bars indicate statistically significant differences among means within each measured parameter at *P* < 0.05, as determined by Duncan’s multiple range test.

Graft compatibility significantly influenced the color attributes of ‘Dinosaur Egg’ pluot fruit, with distinct response patterns observed between the exocarp and mesocarp (**Figure 3**). Regarding the exocarp, a systematic color shift occurred as graft compatibility decreased (**Figures 3A, B**). Fruits from the low-compatibility groups (Grades I, II, and III) exhibited significantly higher values for lightness, yellowness, chroma, and hue angle compared to the control, indicating a brighter, more yellow, vivid, and yellow-shifted exocarp. In contrast, the exocarp color index of ‘Dinosaur Egg’ showed an inverse trend, being highest in the control, which suggests superior red color development in high-compatibility fruit (**Figure 3C**). No significant difference was found in exocarp redness among the groups. Color variation in the mesocarp was more complex. Mesocarp lightness was highest in Grade I. Redness was higher in the control and Grade I, whereas yellowness and hue angle were significantly elevated in Grades II and III. The comprehensive mesocarp color index of ‘Dinosaur Egg’ mirrored that of the exocarp, showing a significant decline with decreasing graft compatibility, with the highest value in the control. No significant inter-group differences were detected for mesocarp chroma. In summary, graft compatibility plays a significant role in regulating fruit coloration. Lower compatibility induced a shift in exocarp color towards brighter and more yellowish hues but concurrently reduced the overall red intensity, as indicated by lower color index of ‘Dinosaur Egg’ values in both the exocarp and mesocarp.

#### 3.1.3 Basic nutritional components and flavor quality

**Figure 4:**
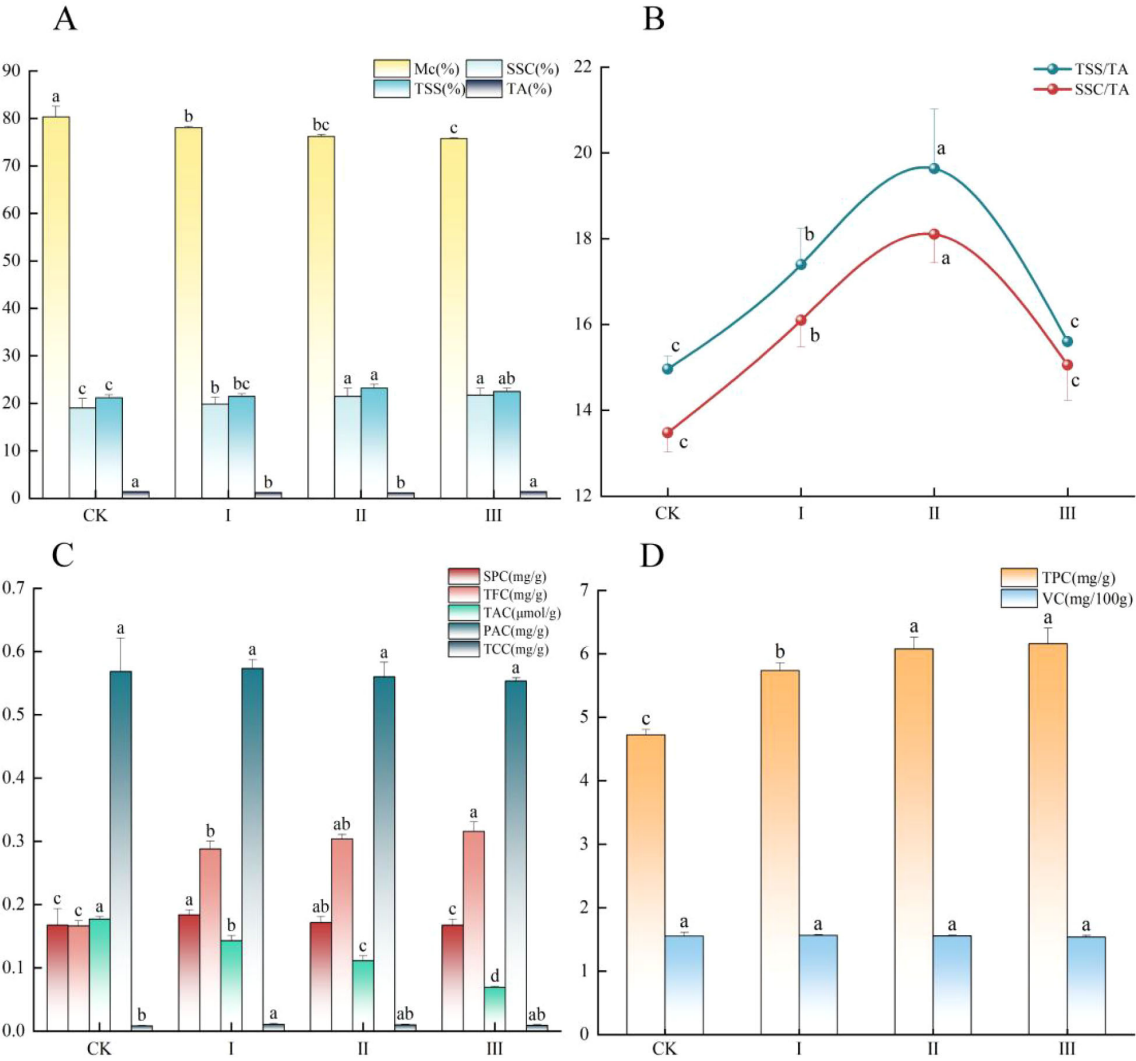
Flavor attributes and bioactive compounds in ‘Dinosaur Egg’ fruits under different graft compatibility grades. **(A)** moisture content (Mc), soluble solids content (SSC), total soluble sugars (TSS) and titratable acidity (TA); **(B)** Sugar-to-acid ratio (TSS/TA) and soluble solids content–to–titratable acidity ratio (SSC/TA); **(C)** Soluble protein content(SPC), total flavonoid content (TFC), total anthocyanin content (TAC), proanthocyanidin content (PAC), and total carotenoid content (TCC); **(D)** Total phenolic content (TPC) and ascorbic acid content (vitamin C, VC). Data are presented as mean ± SE (n = 9). Different lowercase letters (a–d) above bars indicate statistically significant differences among means within each measured parameter at *P* < 0.05, as determined by Duncan’s multiple range test.

Graft compatibility had a significant impact on the accumulation of moisture and flavor-related compounds in ‘Dinosaur Egg’ pluot fruits (*P* < 0.05) (**Figures 4A, B**). Moisture content progressively declined with decreasing graft compatibility, being highest in the control and lowest in Grade III (**Figure 4A**). Conversely, soluble solids content showed a continual increase, reaching its maximum in fruits from Grades II and III (**Figure 4A**). Further analysis of flavor components revealed an inverse relationship between total soluble sugars and titratable acidity (**Figure 4A**). The highest level of total soluble sugars was found in Grade II fruits, whereas titratable acidity peaked in both the control and Grade III fruits. Consequently, the most favorable balance between sweetness and acidity was achieved in the moderate compatibility group (Grade II). The key indices of flavor balance, the TSS/TA and SSC/TA ratios, showed a consistent trend (**Figure 4B**). Both ratios peaked in Grade II fruits and were significantly higher than those in all other groups (*P* < 0.05), indicating an optimal sweet-sour balance. This analysis demonstrates that graft compatibility profoundly shapes sensory quality by differentially regulating the accumulation of moisture, soluble solids, sugars, and acids. Notably, fruits from the Grade II compatibility group maintained relatively high levels of soluble solids and sugars while achieving a superior sugar-acid balance, thereby exhibiting the greatest potential for overall flavor quality.

#### 3.1.4 Bioactive compounds

Both total phenolics and total flavonoids increased significantly with decreasing graft compatibility, attaining the highest concentrations in Grade III fruits, which were significantly greater than those in the control (**Figures 4C, D**). In contrast, total anthocyanins exhibited an inverse trend, declining significantly as compatibility decreased (**Figure 4C**). Their concentration was lowest in Grade III fruits, representing only 39.1% of the control level. Proanthocyanidin content did not differ significantly among the groups. Soluble protein content increased initially then declined with decreasing graft compatibility, peaking in fruits from Grades I and II (**Figure 4C**). A similar trend was observed for carotenoids, with the highest level in Grade I and the lowest in the control (**Figure 4C**). No significant inter-group differences were detected for ascorbic acid content (**Figure 4D**). Collectively, these results demonstrate that graft compatibility directs the accumulation of fruit secondary metabolites. Lower compatibility (Grade III) stimulates the biosynthesis of total phenolics and flavonoids but suppresses the accumulation of anthocyanins. In contrast, moderate compatibility (Grades I and II) favors the enrichment of soluble proteins and carotenoids.

#### 3.1.5 Mineral composition

Graft compatibility significantly altered the mineral composition of ‘Dinosaur Egg’ pluot fruits (*P* < 0.05), with each element exhibiting distinct accumulation patterns across the groups (**Table 2**). Among the macronutrients, B concentration exhibited a continuous increase with decreasing graft compatibility, reaching its peak in Grade III fruits, which was significantly higher than in all other groups. P, Mg, and K displayed similar patterns, accumulating to the highest levels in Grades II and III, significantly exceeding those in the control and Grade I. In contrast, Ca concentration showed an initial decline followed by a rise, being lowest in Grade II and highest in Grade III fruits. The accumulation patterns of trace elements distinctly differed from those of macronutrients. Mn, Fe, Cu, and Zn all attained the highest concentrations in CK fruits and exhibited an overall declining trend with decreasing graft compatibility, reaching their lowest levels in either Grade I or Grade II fruits.

**Table 2:** Mineral composition of ‘Dinosaur Egg’ fruits under different graft compatibility grades (mg·kg^-1^ FW).

Mineral nutrition	CK	I	II	III
B	5.7067 ± 0.3326 d	7.2058 ± 0.2819 c	8.8385 ± 0.1148 b	10.4218 ± 0.1984 a
Mg	75.7206 ± 2.0514 b	69.8554 ± 1.7994 c	86.5155 ± 2.2673 a	87.9479 ± 3.5123 a
P	316.8996 ± 7.4219 b	293.2229 ± 9.4052 b	436.1453 ± 15.3755 a	427.8492 ± 20.4863 a
K	3264.4513 ± 88.5856 b	3172.4006 ± 36.8132 b	3488.8818 ± 98.0249 a	3631.3032 ± 133.8438 a
Ca	11.1863 ± 0.1424 ab	10.9614 ± 0.2081 ab	10.4846 ± 0.1469 b	11.7168 ± 0.9351 a
Mn	0.8054 ± 0.0231 a	0.3882 ± 0.0424 c	0.3633 ± 0.0115 c	0.4565 ± 0.0152 b
Fe	28.3334 ± 2.2851 a	6.9800 ± 0.5112 b	2.7401 ± 0.1507 c	7.8003 ± 0.7592 b
Cu	1.0616 ± 0.0182 a	0.6159 ± 0.0245 d	0.7214 ± 0.0393 c	0.7856 ± 0.0047 b
Zn	1.5935 ± 0.0837 a	0.9648 ± 0.1606 c	1.2109 ± 0.0195 b	1.1414 ± 0.0521 bc

Data are presented as mean ± SD (n = 3). Different lowercase letters (a–d) within the same row indicate statistically significant differences among treatments according to Duncan’s multiple range test at P < 0.05.

Taken together, these patterns demonstrate that graft compatibility differentially regulates the accumulation of mineral elements in fruit. High-compatibility combinations (the control) favored the enrichment of trace elements (Mn, Fe, Cu, Zn), whereas lower compatibility promoted the accumulation of macronutrients, particularly B, P, Mg, and K.

### 3.2 Correlation analysis

#### 3.2.1 Correlations among fruit quality traits

**Figure 5:**
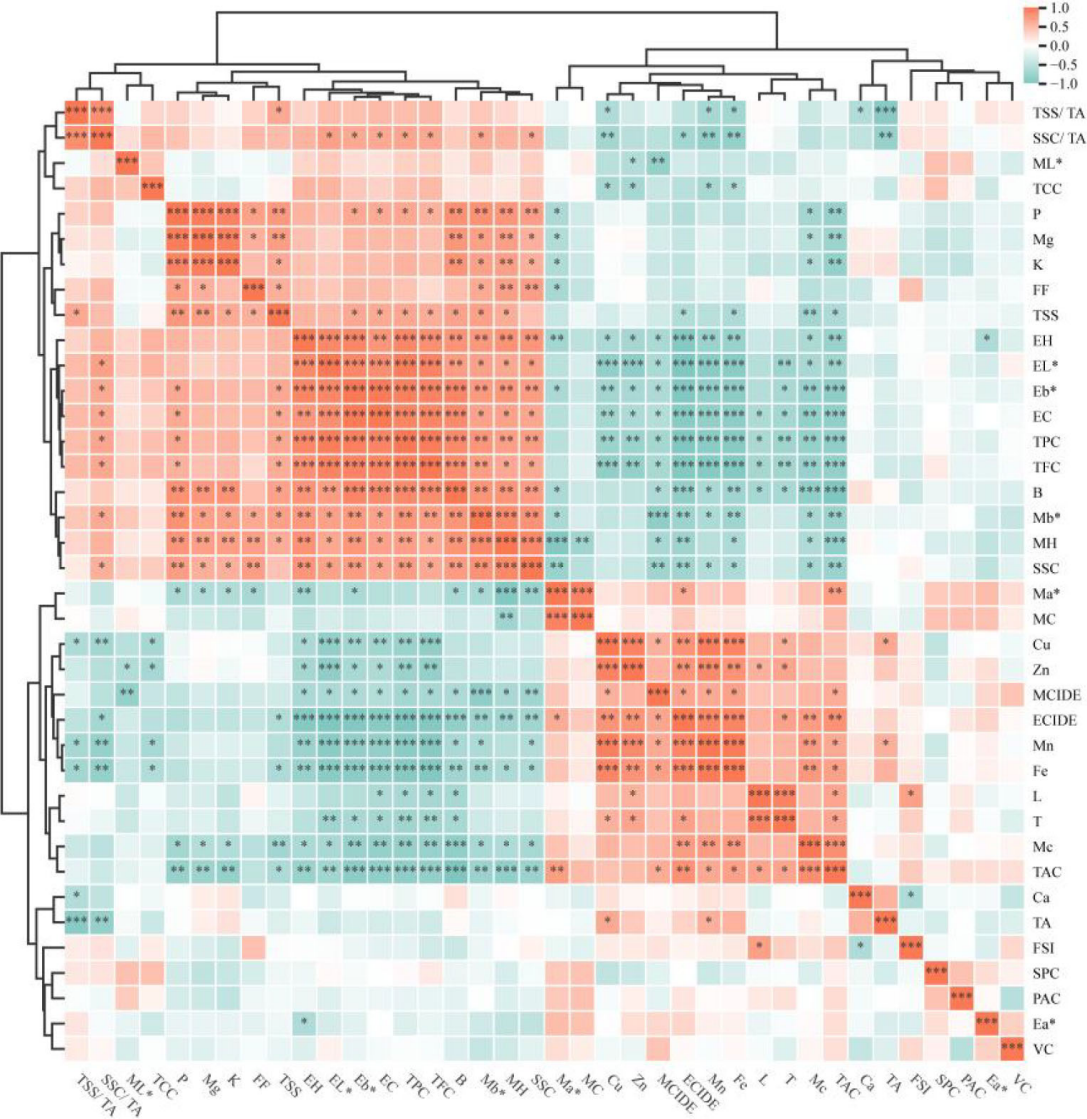
Hierarchical clustering heatmap of Pearson correlations among 39 quality indicators in ‘Dinosaur Egg’ fruit. Red and blue colors indicate positive and negative correlations, respectively. The asterisks *, **, and *** denote statistical significance at the *p* < 0.05, 0.01, and 0.001 levels, respectively.

Pearson correlation analysis of the 39 quality traits in ‘Dinosaur Egg’ pluot fruit revealed close interrelationships, forming a complex network of synergies and trade-offs (**Figure 5**). Flavor attributes were predominantly regulated by acidity. The TSS/TA and SSC/TA ratios exhibited a strong positive correlation (r = 0.84, *p* < 0.001) and were both strongly negatively correlated with titratable acidity (r = -0.91, -0.80; *p* < 0.001). Mineral elements demonstrated modular co-accumulation. The macronutrients P, Mg, and K were strongly positively intercorrelated (r > 0.91, *p* < 0.001). Similarly, the micronutrients Cu, Zn, Mn, and Fe showed extensive positive correlations (e.g., Mn and Fe, r = 0.98, *p* < 0.001). Beyond these inter-element relationships, P exhibited significant correlations with multiple quality traits. It was positively correlated with soluble solids content (r = 0.72, *p* < 0.01) and total soluble sugars (r = 0.76, *p* < 0.01). In addition, P was significantly positively correlated with the yellow–blue value (b*) of both the exocarp (r = 0.64, *p* < 0.05) and the mesocarp (r = 0.82, *p* < 0.01), and significantly negatively correlated with the red–green value (a*) of the mesocarp (r = −0.64, *p* < 0.05). These correlations suggest that P may contribute to a shift in fruit color toward yellow as graft affinity decreases. B played a pivotal role, being strongly positively correlated with P, K, total phenolics, and total flavonoids, but strongly negatively correlated with total anthocyanins (r = -0.98, *p* < 0.001).

Fruit color traits were closely interrelated yet followed distinct pathways. Exocarp lightness and yellowness showed strong positive correlations with total phenolics and total flavonoids, but strong negative correlations with the exocarp color index of ‘Dinosaur Egg’ (r < -0.97, *p* < 0.001), indicating a metabolic trade-off between a “bright yellow” and a “deep red” exocarp. Mesocarp redness and chroma were positively correlated with total anthocyanin content, confirming the contribution of anthocyanins to mesocarp redness. The accumulation of health-promoting compounds occurred via different pathways. Total phenolics and total flavonoids were strongly positively correlated (r = 0.97, *p* < 0.001), but both were strongly negatively correlated with total anthocyanin accumulation. Total anthocyanin content was strongly positively correlated with moisture content (r = 0.84, *p* < 0.001). In addition, fruit firmness was positively correlated with soluble solids content, while fresh weight, longitudinal diameter, and transverse diameter were negatively correlated with the concentrations of certain mineral elements and exocarp lightness.

#### 3.2.2 Screening of key quality indicators

To mitigate the influence of high multicollinearity among the original variables on the objectivity of the subsequent comprehensive evaluation and to establish a more parsimonious and effective evaluation system, the 39 initially measured traits were systematically screened based on the correlation analysis results (Section 3.2.1). The screening procedure adhered to the following principles: (i) removal of redundant variables exhibiting high collinearity (|r| > 0.9); (ii) retention of informative and independent variables; (iii) assurance that all relevant quality dimensions remained represented; and (iv) preservation of core indicators essential for fruit quality assessment.

Based on the aforementioned criteria, 20 key indicators were selected for subsequent analyses: fresh weight (FW), fruit shape index (FSI), exocarp a* (Ea*), mesocarp a* (Ma*), exocarp color index of ‘Dinosaur Egg’ (ECIDE), mesocarp color index of ‘Dinosaur Egg’ (MCIDE), fruit firmness (FF), moisture content (MC), ascorbic acid content (vitamin C, VC), B, P, Ca, Fe, soluble solids content (SSC), total soluble sugars (TSS), titratable acidity (TA), TSS/TA, total phenolics (TPC), total anthocyanins (TAC), and total carotenoids (TCC). This assessment system, comprising twenty indicators, comprehensively captures the multidimensional characteristics of fruit quality while effectively avoiding redundancy among the indicators.

### 3.3 Principal component analysis

Principal component analysis (PCA) was conducted on the 20 key fruit quality traits selected in Section 3.2.2; the eigenvalues, variance explained, and cumulative variance explained of the principal components are presented in **Table 3**. Following standard criteria (eigenvalue ≥ 1 and cumulative variance explained ≥ 85%), the first five principal components were retained. These five components collectively explained 89.07% of the total variance, effectively capturing the primary information in the original dataset.

**Table 3:** Principal component analysis of 20 quality indicators in ‘Dinosaur Egg’ fruits.

Index	Principal component				
	PC1	PC2	PC3	PC4	PC5
FW	0.1778	0.0537	0.3551	-0.0520	0.4981
FSI	0.0477	0.2928	0.4986	-0.0628	-0.2460
Ea*	0.0975	0.1585	-0.3817	0.3868	0.1138
ECIDE	0.3075	-0.0592	0.0809	0.1546	0.1332
Ma*	0.2310	0.1228	-0.3027	-0.0205	0.0430
MCIDE	0.2424	0.0693	0.0083	0.2527	-0.1244
FF	-0.2050	0.0861	0.4051	0.2864	-0.0712
MC	0.2848	0.0116	0.1013	-0.1427	0.0068
VC	0.0604	0.2947	-0.1461	0.1753	-0.5967
B	-0.3054	-0.1699	-0.0933	0.0894	-0.0049
P	-0.2627	-0.0865	0.1172	0.3257	0.1366
Ca	0.0251	-0.4690	-0.1565	-0.0974	0.1366
Fe	0.2920	-0.1624	0.1789	0.1567	-0.0839
SSC	-0.2790	-0.0825	0.2405	-0.1068	-0.1041
TSS	-0.2469	0.1096	0.0519	0.2628	0.2244
TA	0.0761	-0.4695	0.1278	0.0908	-0.2529
TSS/TA	-0.1600	0.4246	-0.0748	0.0706	0.3037
TPC	-0.3147	0.0462	-0.1276	-0.0758	-0.1297
TAC	0.3013	0.1915	0.0736	-0.0981	0.0560
TCC	-0.1223	0.1657	-0.0510	-0.6044	-0.0226
Eigenvalues	9.15	3.29	2.26	1.80	1.31
Variance explained (%)	45.77	16.46	11.32	8.98	6.56
Cumulative variance explained (%)	45.77	62.22	73.54	82.51	89.07

Values represent the component loadings for each indicator on principal components 1–5 (PC1–PC5). The asterisk (*) in the superscripts of Ea* and Ma* indicates the color parameter (red–green chromaticity a*) for exocarp (E) and mesocarp (M), respectively.

Principal Component 1 (PC1) was strongly associated with the exocarp color index, total anthocyanins, and Fe, representing fruit color attributes. This component was therefore designated as representing color quality. PC2 showed a strong positive correlation with the TSS/TA ratio and a strong negative correlation with titratable acidity, thereby capturing fruit flavor profiles. Accordingly, PC2 was assigned to represent flavor quality. PC3 was strongly associated with the fruit shape index, fruit firmness, and fresh weight, reflecting the physical appearance of the fruit. This component was thus defined as representing appearance quality. PC4 is mainly associated with mineral elements, especially P, Fe, and B. Therefore, PC4 is designated as representing mineral nutrition. PC5 is closely associated with ascorbic acid, total phenolic content, and total anthocyanins. Therefore, PC5 is designated as representing bioactive compounds. In summary, the 20 fruit quality traits were categorized into five distinct dimensions: color quality, flavor quality, appearance quality, mineral nutrition, and bioactive compounds.

### 3.4 Comprehensive evaluation of fruit quality across graft compatibility groups

Based on the membership function method and dimensional scoring, the composite evaluation scores and the dimensional scores were calculated for the four graft compatibility groups of ‘Dinosaur Egg’ pluot (**Table 4**). The composite evaluation score revealed significant differences in overall fruit quality among the groups. The ranking order was: Grade II > CK > Grade III > Grade I. Specifically, Grade II achieved the highest composite score (0.7233), indicating superior overall quality, whereas Grade I yielded the lowest score (0.6577).

**Table 4:** Results of the comprehensive quality evaluation for ‘Dinosaur Egg’ fruits under different compatibility grades.

Graft compatibility grade	Color quality	Flavor quality	Appearance quality	Mineral nutrition	Bioactive compounds	Composite evaluation score	Rank
II	0.1302	0.1920	0.0581	0.1830	0.1599	0.7233	1
CK	0.1879	0.1100	0.0638	0.1746	0.1446	0.6810	2
III	0.1192	0.1605	0.0481	0.2008	0.1379	0.6664	3
I	0.1486	0.1463	0.0543	0.1365	0.1720	0.6577	4

Analysis of the dimensional scores revealed distinct patterns among the groups. Although Grade II fruit scored highest in flavor quality, it maintained a relatively balanced performance across the other four dimensions. The control fruit excelled in appearance quality and color quality but received the lowest score for flavor quality. Grade III fruit peaked in mineral nutrition yet scored lowest in appearance quality, color quality, and bioactive compounds. Grade I fruit achieved the highest score in bioactive compounds and the lowest in mineral nutrition, with unremarkable performance in the remaining three dimensions.

## 4 Discussion

### 4.1 Regulation of appearance quality by graft compatibility

Appearance quality is a fundamental determinant of fruit market value. The present findings indicate that graft compatibility influences fruit appearance primarily by modulating fruit growth rather than altering its inherent morphological traits. Specifically, lower graft compatibility significantly reduced fresh weight, longitudinal diameter, and transverse diameter. This reduction can be primarily ascribed to localized graft incompatibility, which impairs vascular connectivity between the rootstock and scion, leading to diminished efficiency in water and photoassimilate transport and, consequently, limiting fruit expansion and biomass accumulation (Rasool et al., 2020). This mechanistic explanation has been validated in incompatible combinations of various fruit species, such as peach and plum, where trait manifestation is often delayed and cannot be remedied by introducing a compatible interstock (Ur Rehman et al., 2025). Our results align with numerous reports demonstrating that low-compatibility rootstocks reduce fruit weight in crops such as melon (Camalle et al., 2023), eggplant (Musa et al., 2020), and cucumber (Aslam et al., 2020).

Graft compatibility did not significantly influence the fruit shape index. This observation supports the classical perspective that fruit shape is predominantly governed by genetic factors, whereas final size is modulated by environmental and physiological conditions (Zhang et al., 2022; Glišić et al., 2023). Consistent with this, studies on crops such as avocado (Cano-Gallego et al., 2023) and watermelon (Nie and Wen, 2023) have shown that while rootstocks can alter fruit size, they exert no significant effect on the fruit shape index. Discrepancies exist among studies regarding the impact of compatibility on fruit size (Fredes et al., 2017; Martins et al., 2021). These inconsistencies may originate from the lack of a unified definition for “graft compatibility” itself (Cano-Gallego et al., 2023), coupled with the inherent specificity of rootstock-scion interactions.

Compatibility induced a systematic reprogramming of fruit coloration (MaChado et al., 2015; Chen et al., 2024). In this study, a lower color index of ‘Dinosaur Egg’ fruit corresponds to a more yellow hue, whereas a higher index indicates a redder fruit (Carreño et al., 1995). Reduced compatibility resulted in increased lightness, yellowness, and hue angle in both the exocarp and mesocarp, concomitant with a decreased color index. This suggests that lower compatibility shifts the pigmentation metabolism from an anthocyanin (red/purple)-dominant to a carotenoid (yellow/orange)-accumulating pathway (Wang et al., 2025c). This shift likely arises as incompatibility stress signals simultaneously repress key genes for anthocyanin biosynthesis and activate the carotenoid synthesis pathway (Zhong et al., 2022). Notably, fruits from the Grade II (moderately low compatibility) treatment exhibited not only a bright yellow hue but also the most stable coloration, offering a novel avenue for the targeted breeding of pluots with uniform and visually appealing appearance through strategic rootstock–scion selection.

### 4.2 Bidirectional effects of graft compatibility on internal quality

The internal quality of fruit dictates its sensory and nutritional attributes. Regarding flavor composition, the restricted water transport associated with lower graft compatibility induced a “concentration effect”, leading to significant increases in soluble solids content and total soluble sugars (Ho et al., 1987; Jukić Špika et al., 2021; Gong et al., 2022). Concomitantly, titratable acidity displayed a non-monotonic pattern. It decreased from the control to Grade II, which optimized the TSS/TA and SSC/TA ratios. However, a rebound occurred under the severe incompatibility of Grade III, disrupting the flavor balance. This highlights that moderate stress is crucial for flavor enhancement, whereas excessive incompatibility may impair sugar metabolism (Camalle et al., 2023). They also align with previous studies in other cucurbit crops. Correlation analysis revealed positive associations between P, Mg, and K and both total soluble sugars and soluble solids content. This suggests that lower graft compatibility may indirectly promote sugar accumulation and metabolic activity in fruit by enhancing root uptake and translocation of these key mineral elements (Zhu et al., 2020). Furthermore, fruits from low-compatibility combinations exhibited significantly higher fruit firmness, a finding consistent with previous reports (Davis et al., 2008; Kyriacou and Soteriou, 2015; Alan et al., 2017; Trandel et al., 2021). This increased firmness is often associated with alterations in cell wall architecture or the accumulation of osmoregulatory compounds (e.g., citrulline) (Soteriou et al., 2017; Liu et al., 2025). While beneficial for postharvest handling and storage (Martins et al., 2021), elevated firmness may influence consumer preferences regarding fruit texture.

Regarding functional components, decreased graft compatibility markedly activated secondary metabolic defense responses, leading to the enhanced accumulation of total phenolics and total flavonoids (30.4% increase in total phenolics in Grade III compared to CK) (Shivran et al., 2022). However, a significant resource trade-off in metabolic flux was observed. Under low-compatibility conditions, the biosynthesis of total anthocyanins was specifically suppressed (with Grade III content being only 39.1% of the control), and the accumulation of soluble proteins was also inhibited (Camalle et al., 2023). In our study, total anthocyanin content was strongly positively correlated with moisture content but negatively correlated with total phenolics and flavonoids. This suggests that the water stress induced by low compatibility likely redirects metabolic flux away from anthocyanin biosynthesis towards the synthesis of other phenolic compounds (Musa et al., 2020; Popova et al., 2023; Camalle et al., 2023). This pivotal metabolic trade-off fundamentally reshapes the functional composition of fruit under low-compatibility conditions. Notably, neither ascorbic acid content nor proanthocyanidin content varied significantly across treatments, indicating that their biosynthesis is primarily genetically programmed and remains relatively unresponsive to graft compatibility-induced stress (Parisi et al., 2023).

### 4.3 Selective regulation of fruit mineral nutrition by graft compatibility

Mineral elements constitute the fundamental basis for fruit quality development and serve as an essential source of nutrients in the human diet (White and Broadley, 2009). This study confirmed that graft compatibility profoundly reshaped the mineral accumulation profile in ‘Dinosaur Egg’ pluot fruit; reduced compatibility markedly elevated the fruit concentrations of B, P, Mg, and K (Nawaz et al., 2016). Notably, B concentration in Grade III (lowest compatibility) fruit increased substantially by 82.6% relative to the control, underscoring its central role as a hub integrating distinct quality pathways. This aligns with its pivotal functions in cell wall synthesis, sugar transport, and phenolic metabolism (Wang et al., 2025c). Furthermore, these elements exhibited strong inter-correlations, indicative of a coordinated uptake and translocation mechanism (Habibi et al., 2022). The accumulation of P, K, and Mg, which are core elements for energy metabolism, enzymatic activation, and osmotic regulation, provided the necessary substrates and energy for the synthesis of sugars and phenolic compounds (Cai et al., 2012). The synchronized increase in these elements under low compatibility may reflect a root-level adaptive response, potentially involving altered expression of mineral transporters (Di Gioia et al., 2017), though direct molecular evidence is needed for confirmation. This finding aligns with previous reports that low-compatibility rootstocks enhance accumulation of P, K, and Ca in tomato (Gong et al., 2022) and cucumber fruits (Noor et al., 2019).

In contrast, the concentrations of trace elements, including Fe, Mn, Cu, and Zn, showed a significant declining trend with decreasing graft compatibility. For example, the Fe concentration in the control fruits was 10.3-fold higher than in Grade II fruits. This decline may be attributed to two factors. First, a nutrient antagonism effect, where high levels of P and K could inhibit the availability of other elements (through the formation of insoluble iron phosphates) (Xie et al., 2021). Second, correlation analysis revealed strong positive correlations among these trace elements, suggesting that their uptake may be co-regulated by a shared transport system—whose function could be selectively inhibited at the low-compatibility graft union (Nawaz et al., 2016; Pina et al., 2017). However, the exact molecular mechanisms underlying this selective impairment warrant further investigation. Notably, Ca accumulation exhibited a distinct pattern when expressed on a fresh weight basis, with its concentration in Grade III fruits (11.72 mg·kg^-1^ FW) being comparable to that of the control group (11.19 mg·kg^-1^ FW). Although this appears counterintuitive given the expected vascular disruption in incompatible grafts, this phenomenon can be explained by a concentration effect resulting from reduced fruit moisture content under severe incompatibility stress. Our data showed that fruit moisture progressively decreased from 80.32% in the control to 75.75% in Grade III. This 4.57% reduction from CK to Grade III would be expected to concentrate minerals, including calcium, on a fresh weight basis. When expressed on a dry weight basis to account for this moisture difference, the calcium content in Grade III (48.33 mg·kg^-1^ DW) was lower than that in the control group (56.86 mg·kg^-1^ DW), which was in complete accordance with the expectation of impaired calcium transport in the incompatible combination.

In summary, rootstock affinity may selectively regulate the longitudinal transport of mineral elements by altering root physiology, vascular connectivity at the graft junction, and the expression of nutrient transporters. This selective control not only directly determines the mineral composition of the fruit, but also indirectly and profoundly influences its overall quality by supplying substrates for specific metabolic pathways.

### 4.4 Determining the optimal compatibility threshold for fruit quality

A comprehensive evaluation using membership function analysis showed that fruits from the Grade II compatibility treatment achieved the highest composite score. This result reveals a new pattern—that optimal overall fruit quality does not necessarily arise from full compatibility—thereby offering a novel perspective for rootstock and scion selection that goes beyond the conventional “compatibility-first” approach.

Different graft compatibility grades shaped characteristic quality profiles, presenting a distinct “trade-off map.” The control showed the best performance in appearance and color, consistent with its higher moisture content and total anthocyanin accumulation (Rampáčková et al., 2021), yet it received the lowest flavor quality score due to a relatively bland TSS/TA (total soluble solids to titratable acidity) ratio. Fruits from the Grade II compatibility treatment achieved the highest flavor quality score, attributable to their optimal sugar-acid balance and higher soluble solids content, aligning with reports that lower affinity can enhance flavor (Colla et al., 2006; Jukić Špika et al., 2021; Gong et al., 2022). Crucially, this treatment maintained consistently high and balanced levels across the other four quality dimensions without major deficiencies, leading to the optimal overall quality. In contrast, Grade I and Grade III compatibility exhibited unbalanced, extreme profiles: Grade I excelled in bioactive compounds but scored the lowest in mineral nutrition; whereas Grade III, despite attaining the highest mineral nutrition score, severely compromised other dimensions such as appearance and color, ultimately reducing the comprehensive fruit quality.

Based on the comprehensive evaluation (**Table 4**) and mineral composition data (**Table 2**), the optimal compatibility threshold (Grade II) was associated with specific alterations in fruit mineral content. Compared to the control, Grade II fruits exhibited moderate reductions in trace elements, with Fe decreasing by 90%, Mn by 55%, Cu by 32%, and Zn by 24%, as well as a slight decrease in Ca (6%). Conversely, macronutrients including B, P, K, and Mg increased by 55%, 38%, 7%, and 14%, respectively. These quantitative changes indicate that such reductions in trace elements can be considered acceptable when accompanied by enhanced accumulation of key macronutrients and improved flavor and bioactive compound profiles. This trade-off underscores that moderate incompatibility can achieve an optimal balance across multiple quality dimensions, providing a practical reference for rootstock selection.

These findings are consistent with the concept of hormesis, a phenomenon in which moderate, non-lethal stress can trigger adaptive metabolic responses that optimize physiological output (Colla et al., 2006; Shivran et al., 2022). In the present grafting system, we propose that moderate low-affinity stress, while slightly inhibiting the transport of water and photoassimilates (leading to a marginal reduction in individual fruit weight) (Rasool et al., 2020), may serve as a signaling cue that triggers the following adaptive adjustments: (i) promoting the targeted accumulation of sugars, specific phenolic compounds (total phenolics, total flavonoids), and key mineral elements (B, P, K, Mg) (Evrenosoğlu et al., 2010; Zhu et al., 2020); (ii) optimizing the sugar-acid ratio via a “concentration effect”, thereby significantly enhancing flavor quality (Ho et al., 1987; Gong et al., 2022); and (iii) reallocating metabolic resources within an acceptable range. Ultimately, this process achieves an optimal balance across multiple quality dimensions rather than maximizing any single trait. However, confirming a true hormetic response requires direct measurements of stress indicators (e.g., ROS levels, antioxidant enzyme activities, ABA) and dose-response relationships, which were beyond the scope of this study. Therefore, we propose this explanation as a working hypothesis for further research.

In rootstock selection and grafting practice, exclusive pursuit of theoretical full compatibility is not imperative. Instead, the proactive screening and utilization of rootstock-scion combinations that can stably induce a moderate, controllable low-affinity stress may serve as an innovative cultivation strategy. This approach allows for the targeted and synergistic improvement of fruit flavor, nutritional quality, and health-promoting value (e.g., via specific antioxidant compounds). Future work should focus on elucidating the molecular mechanisms underlying the “moderate stress” regulatory network. Furthermore, multi-site and multi-year trials should be undertaken to establish an “optimal compatibility threshold” database for major scion cultivars, thereby enabling the precise design and application of rootstocks for premium fruit production.

## 5 Conclusion

This study demonstrates that for the ‘Dinosaur Egg’ fruit, moderate low graft compatibility (Grade II), rather than full compatibility, optimizes overall fruit quality. Reduced compatibility inhibited fruit size and shifted pericarp color from red to yellow, while concurrently elevating soluble solids and sugar content via a “concentration effect.” Notably, moderate low compatibility (Grade II) achieved an optimal sugar-acid ratio, resulting in the best flavor profile. Regarding bioactive compounds, reduced graft compatibility triggers secondary metabolic defense responses, leading to a significant increase in the accumulation of total phenolics and flavonoids while concurrently suppressing anthocyanin biosynthesis. Furthermore, graft compatibility differentially modulated mineral nutrition: low affinity promoted the accumulation of B, P, K, and Mg but suppressed the translocation of Fe, Mn, Cu, and Zn. As revealed by comprehensive evaluation and mineral composition analysis, the optimal compatibility threshold (Grade II) exhibited moderate reductions in Fe (90%), Mn (55%), Cu (32%), Zn (24%), and Ca (6%), accompanied by enhanced macronutrient accumulation and improved flavor and bioactive profiles, indicating that such trade-offs are acceptable for superior fruit quality. In summary, graft compatibility can influence fruit quality by modulating resource allocation. The utilization of rootstock–scion combinations capable of inducing stable, moderately low-affinity stress may represent a novel approach for the targeted improvement of fruit quality and provide important insights for precision horticulture.

## Data Availability

The raw data supporting the conclusions of this article will be made available by the authors, without undue reservation.
